# The European Society of Cardiology 2024 Guidelines on Chronic Coronary Syndromes: A Critical Appraisal

**DOI:** 10.3390/jcm14041161

**Published:** 2025-02-11

**Authors:** Roberto Ferrari, Luis Henrique W. Gowdak, Francisco Padilla, David K. L. Quek, Saumitra Ray, Giuseppe Rosano, Ciro Indolfi, Pasquale Perrone Filardi

**Affiliations:** 1 Department of Cardiology, University of Ferrara, 44121 Ferrara, Italy; 2 Centre of Prevention, Corso Ercole I D’Este 32, 44121 Ferrara, Italy; 3 Atherosclerosis and Chronic Coronary Artery Disease Unit, Heart Institute, Sao Paulo 05403-010, Brazil; 4 Cardiología Clínica e Intervencionista Tarascos, Guadalajara, Mexico; 5 Pantai Hospital Kuala Lumpur, Kuala Lumpur 59100, Malaysia; drquek@gmail.com; 6 Woodlands Hospital, Kolkata 700027, India; drsaumitra@yahoo.co.in; 7 Vivekananda Institute of Medical Sciences, Kolkata 700026, India; 8 Clinical Academic Group, St George’s Hospitals NHS Trust, Blackshaw Road, London SW17 0QT, UK; 9 Department of Medical and Surgical Sciences, Magna Græcia University of Catanzaro, 88100 Catanzaro, Italy; 10 Department of Advanced Biomedical Sciences, Federico II University of Naples, Via Pansini, 5, 80131 Naples, Italy; 11 Mediterranea Cardiocentro, Via Orazio, 2, 80122 Naples, Italy

**Keywords:** chronic coronary syndrome, myocardial ischaemia, angina, treatment, diagnosis, microvascular angina, ANOCA, INOCA

## Abstract

**Background**: During the 2024 annual meeting in London, The European Society of Cardiology released new guidelines (*GLs*) on chronic coronary syndromes (*CCSs*) and simultaneously published them in the European Heart Journal. **Method**: A few experts on the topic from Europe, South America, India, and Asia, who attended the presentation and the Question and Answer sections, met virtually to comment on the GLs after carefully reading the 123-page document. **Result**: There is a consensus that the presented GLs are a comprehensive, up-to-date, clear document of the available data on how to diagnose and treat CCSs and a definite step forward compared to all previous GLs. Of particular value are (a) the efforts to link both diagnosis and treatment to the underlying pathophysiology with the recognition that not all the ischaemic episodes are the same; (b) the decision to adopt the graphic of the so-called “*Diamond Approach*”, although its spirit that no antianginal drug is superior to another is not fully adopted; and (c) the innovative way it condenses and expresses the relevant messages with eye-catching illustrations. **Conclusions**: The present article summarises and comments on the 123-page GLs, highlighting strengths and weaknesses according to the thoughts of the authors.

## 1. Introduction

The European Society of Cardiology (ESC) released the **Guidelines on Chronic Coronary Syndromes** during its annual congress in London in 2024 and published them simultaneously in the *European Heart Journal* [1]. This comprehensive document spans 123 pages, accompanied by an additional 12 pages of supplementary data and enhanced with illustrations that complement the traditional ESC tables. A concise version, called the “Pocket Guidelines”, is also available.

The objectives of this brief review are threefold.

First, we want to commend the efforts of the 28 members of the Task Force. Producing new guidelines without fresh data on treatments—a focus that often (and mistakenly) captures the attention of many cardiologists—is challenging. Notably, the 2024 guidelines shift the emphasis away from treatment and towards understanding the pathophysiology of chronic coronary syndromes. The guidelines acknowledge that not all ischaemic episodes are identical, underscoring the need for specific diagnoses and, ultimately, tailored treatments.

It is refreshing to note that these guidelines stress the need to understand that chest pain syndromes are not all treatable by coronary intervention, which has dominated the mindsets of the current generation of cardiologists. While percutaneous coronary intervention plays an important role in many myocardial ischaemic syndromes, non-obstructive coronary pathologies and their resultant angina in chronic coronary syndromes have been brought into better focus.

Another unique feature of these GLs is to include patient representatives and methodologists in the Task Force along with experts from the medical field. Thus, in many sections, patient choices were given importance, which emphasises that individual choices in decision-making are important.

Second, we comment on adopting the graphic from the previously published “Diamond Approach” [2]. Lastly, we discuss various aspects of the guidelines, highlighting areas where improvements could still be made. Let us delve into the details.

## 2. Preamble to the ESC Guidelines

This paper serves as a concise overview of concepts related to coronary artery disease, particularly the need to move beyond the outdated idea of fixed, focal, flow-limiting atherosclerotic stenosis in large coronary arteries as the sole cause of myocardial ischaemia and angina. It is essential to recognise that there are various sources of myocardial ischaemia and, consequently, angina.

In addition to the well-known causes of angina—such as anaemia, hypertension, tachycardia, myocardial hypertrophy, myocardial bridging, congenital abnormalities of the coronary arteries, and vasospasm—new evidence now supports the existence of previously suspected functional and/or structural abnormalities in the microcirculation [3].

The contemporary terminology of ANOCA (angina with non-obstructive coronary arteries) and INOCA (ischaemia with non-obstructive coronary arteries) is embraced, highlighting these conditions as prevalent topics within the text. This very differentiation between ANOCA and INOCA hints at the fact that ischaemia and angina do not go hand in hand, the former being an objective parameter to document and quantify, while the latter is a subjective area influenced by various outside factors like one’s psychosocial, economic, and attitudinal differences among others.

The document outlines five different clinical presentations and discusses the current epidemiology of chronic coronary syndromes. All this information is captured in a compelling central illustration. However, the tables labelled “What is New” are excessive, with 40 tables containing 139 different classes of recommendations and levels of evidence, making it challenging to remember them all. This can feel like an overwhelming and unnecessary task.

Despite the extensive text and 1211 references included to ensure comprehensiveness, we recommend focusing on the most relevant tables that pertain to the reader’s specific topic of interest.

## 3. Diagnosis

This section is the most innovative and exciting. Four steps are suggested to not only diagnose angina or ischaemia but, more importantly, to identify or at least attempt to determine the underlying causes of the disease.

The first step involves assessing the likelihood of a patient being affected by chronic coronary syndromes linked to the type, severity, and quantity of risk factors, comorbidities, and symptom characteristics. These symptoms are not limited to chest pain or dyspnoea, whether at rest or during exercise, as they can also be considered equivalents to ischaemic pain. Emphasis is placed on thorough anamnesis, which can help understand the underlying physiopathology. Recent data suggest that only 25% of patients suspected of having angina exhibit the classical “textbook” pain with identifiable aggravating and relieving factors [4]. When this typical pain is present, it is highly indicative of ischaemia caused by obstructive coronary artery disease. However, the remaining patients often present with more subtle and less characteristic symptoms that may involve unusual locations and varying precipitating or relieving factors.

Historically, these symptoms were often mistakenly classified as atypical or non-cardiac pain. Today, exploring every aspect of symptomatology is crucial, as it may reveal microvascular dysfunction. Additionally, it is essential to note that the absence of symptoms does not equate to the lack of ischaemia, particularly in diabetic or elderly patients who lead a sedentary lifestyle. The presence of asymptomatic coronary artery disease is expected, as shown by various surveys and opportunistic screenings [5,6]. The guidelines dedicate a section to this unusual presentation, which, when suspected, should be evaluated using symptom scoring, including a calcium score and, if necessary, coronary computed tomography angiography (CCTA) [1].

A thorough medical history is the first step in determining the subsequent steps in evaluating a patient with suspected coronary artery disease. When typical chest pain is suspected, it is recommended to proceed with a calcium score measure to enhance the likelihood of detecting obstructive coronary artery disease. Following that, CCTA is advisable, and fractional flow reserve (FFR) should also be included, if available. This approach is preferred over traditional exercise electrocardiogram (ECG) testing, which has a lower diagnostic power than CCTA, does not visualise the coronary anatomy, and may be ineffective in concomitant pathologies, such as left bundle branch block or paced rhythm. However, the guidelines recognise that exercise ECG has been the gold standard for diagnosing chronic coronary syndromes for many years. It is cost-effective, widely accessible, and free of radiation. Therefore, exercise ECG remains a valuable tool for detecting ischaemia due to epicardial lesions when CCTA is unavailable, and it can also be beneficial for follow-up assessments.

In cases where the pain does not present typical characteristics, it is reasonable to suspect microvascular or vasospastic angina (ANOCA). In such situations, functional imaging is preferred over anatomical imaging or exercise ECG to confirm the presence of ischaemia. The guidelines provide a comprehensive array of functional tests, including stress echocardiography, myocardial perfusion scintigraphy with single-photon emission computed tomography (SPECT), positron emission tomography (PET), or cardiac magnetic resonance imaging (CMR). The advantages and disadvantages of each test are thoroughly addressed in the accompanying text and tables [1].

Stress echocardiography is the most widely available and utilised method to detect ischaemia. Various techniques to induce ischaemia, including intravenous dobutamine, vasodilators (such as adenosine or dipyridamole, when available), and exercise (if feasible), are discussed in detail. Regardless of the stressor used, stress echocardiography offers several benefits, such as availability, affordability, safety, lack of radiation, and the ability for immediate interpretation by cardiologists, with the option for repeat testing if necessary. However, there are limitations, notably poor image quality in obese patients or those with chronic obstructive pulmonary disease. The guidelines recommend using ultrasound contrast agents (microbubbles) to improve image quality and sensitivity.

Additionally, since stress echocardiography is highly operator-dependent, it may lack reproducibility. If microcirculation dysfunction is suspected, coronary flow reserve (CFR) in the left anterior descending artery (LAD) can be assessed using Doppler flow velocity recordings at rest or during stress. However, the results may vary significantly between operators. More comprehensive measurements of microvascular blood flow (MBF) and reserve (MFR) can be obtained through PET, which measures the increase in blood flow and the capacity of microcirculation to dilate in response to adenosine or regadenoson-induced vasodilation. The guidelines also suggest emerging, although complex and experimental, methods for non-invasive assessment of coronary microvascular dysfunction.

Despite these promising alternatives, the gold standard for diagnosing INOCA or ANOCA remains invasive. The guidelines summarise several invasive functional tests, providing a complete evaluation of coronary physiology. It starts with confirming the absence of significant epicardial lesions while simultaneously measuring CFR, the index of microcirculation resistance (IMR), or hyperaemic myocardial velocity resistance (HMR) to identify endothelial-independent vasodilation abnormalities in the microcirculation. Conversely, endothelial-dependent abnormalities can be assessed through the injection of a low dose of acetylcholine, which can also reveal abnormal vasoconstriction—spasm—in either the epicardial (high-dose acetylcholine) or microvascular (low-dose acetylcholine) coronary artery [1].

Invasive coronary angiography (step 4) is indicated for patients with a very high probability of obstructive stenosis, even after optimised clinical therapy. According to the previous guidelines, invasive assessment is warranted when CCTA indicates severe stenosis in the left main or proximal LAD artery or cases of disease affecting two or three major vessels. Even with the presence of epicardial stenosis, the guidelines recommend measuring FFR or iFR to assess ischaemia or microvascular damage and guide decisions on which areas of the heart to revascularise. The guidelines strongly endorse intravascular imaging through intracoronary ultrasound (IVUS) or optical coherence tomography (OCT) to evaluate the severity of epicardial stenosis.

The proposed diagnostic algorithms for suspected epicardial stenosis or microvascular alterations are comprehensive, current, and innovative. A notable strength is the effort to uncover the underlying pathophysiology of chronic coronary syndrome. Additionally, the guidelines committee deserves commendation for acknowledging that these algorithms may only sometimes be feasible due to complexity and costs. One may question whether all four steps are necessary. The answer is yes. Theoretically, they are essential to justify the need for revascularisation through angioplasty, a therapeutic intervention not associated with a prognostic benefit.

In other words, coronary interventions by angioplasty, for instance, are not mandatory for every patient with chronic coronary syndrome, as they once were. They are required only when they are strictly necessary based on pathophysiology (the presence of ischaemia associated with an obstructive lesion) and the persistence of symptoms not adequately controlled by optimal antianginal therapy.

It is interesting to note that the GLs are both in favour of invasive treatment but also urge caution regarding indiscriminate intervention which, in the past, reached the symbolic record of implanting 67 stents in a single patient as a result of a serial stent-after-stent approach [7]. On the one hand, these GLs reserve the indication of invasive coronary angiography (CAG) to patients with a high possibility of epicardial stenotic lesions when they fail to respond symptomatically to pharmacotherapy. On the other hand, they encourage ad hoc revascularisation at the same sitting when the CAG shows a significant lesion that is proven to be physiologically significant [8].

The 2024 guidelines emphasise patient engagement and informed shared decision-making [9,10,11].

**Figure 1:**
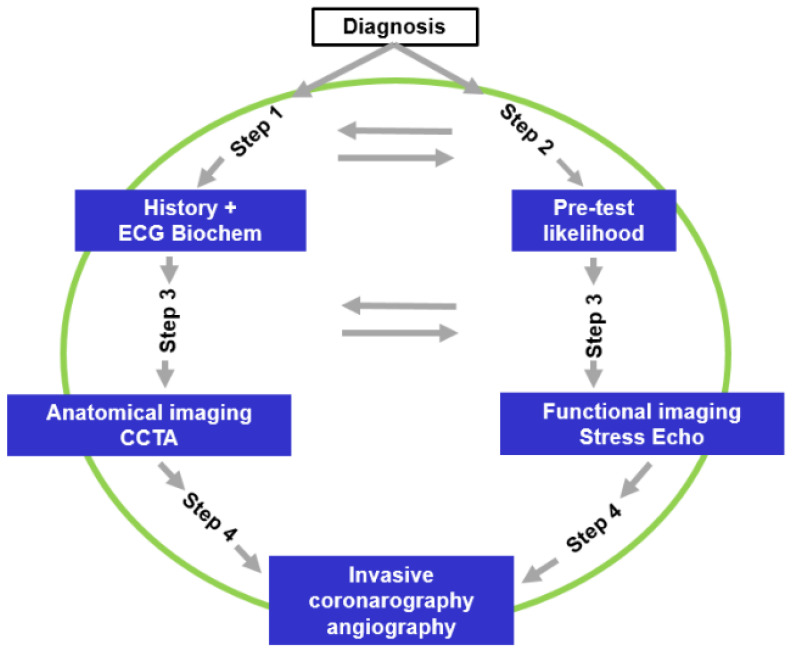
Oversimplified scheme for diagnosis of CCS considering 4 different steps.

We recommend being more aware of the various diagnostic options and practices according to what is accessible and sustainable. If applying the algorithm to all patients is impossible, it should be reserved for the most complex and uncertain cases. Others can often be identified from step 1 through a thorough analysis. For the many patients who cannot access the most advanced diagnostic tools, it is crucial to understand the suggested guidelines for diagnostic escalation and refer those with uncertain diagnoses to specialised centres equipped with the necessary resources before proceeding to unnecessary revascularisation. Figure 1 shows a much simpler scheme for non-specialised centres with limited resources. Step 1 consists of an accurate history with emphasis on the presence of pain and/or dyspnoea plus 12-lead ECG and biochemistry. Possible suspicion of the presence of ischaemia should be considered in light (*step 2*) of the reported new pre-test probability (*1*). If available, measurement of the coronary artery calcium score may be useful.

Symptomatic or even asymptomatic patients with a suspicion (*either low*, *medium*, *or high*) of CCR need to progress to step 3, which is aimed at confirming the presence of ischaemia with either (a) anatomical imaging with CCTA or (b) functional imaging with stress echocardiography using the most available and usual stressor. If possible (*and affordable*), commercial microbubbles are indicated to enhance diagnostic precision. Other forms of imaging, such as CMR and SPECTS, are important if ANOCA is suspected and should be used in highly specialised centres. Invasive coronary angiography, which constitutes the last step (*step 4*), is for patients considered at very high risk, and when the need for revascularisation or the necessity to explore, in detail, the coronary pathophysiology for microvascular damage or vasospasm is envisaged.

In summary, the guidelines provided a precise, up-to-date, innovative, and comprehensive section according to the most recent literature [10,11,12,13].

## 4. Therapy

Chronic coronary syndrome treatment should be viewed holistically, incorporating interventions that aim to (a) slow the progression of coronary atherosclerosis through risk factor management, which includes lifestyle modifications and pharmacotherapy, and (b) alleviate symptoms using appropriate medications or procedures. The central theme of the guidelines is particularly evident in the treatment section, especially regarding symptom management. It is important to note that the same medication is not suitable for all symptoms; instead, the choice of treatment should be based on the underlying pathophysiology and any coexisting health conditions.

- Risk Factor Control and Event Prevention

This section goes beyond the usual simple list of classical risk factors. The guidelines emphasise promoting long-term adherence to lifestyle changes. Achieving this goal can be facilitated through improved interactions between healthcare professionals and patients and through educational programs—single-discipline and multidisciplinary. Additionally, mHealth (mobile health), telehealth, patient-reported outcome measures (PROMs), and feedback play essential roles. The intelligent risk core proposed by the European Association of Preventive Cardiology also contributes to this objective [14].

Two helpful figures summarise these suggestions and outline five actions related to adherence, which are viewed as a form of therapy, along with the use of fixed-dose combination pills. Wearable devices, the internet, and smartphones can positively encourage healthy behaviours.

Attention is given to smoking cessation, including the risks associated with electronic cigarettes and passive smoking, and weight management based on body mass index (BMI). Pharmacological treatment is recommended when diet and regular exercise (with guidance on ideal frequency and intensity) do not achieve the target outcomes. The 2024 guidelines advance the recommendations provided in previous versions.

New classes of medications have been developed that safely promote weight loss and, in turn, reduce the incidence of cardiovascular events, such as cardiovascular death, myocardial infarction, or stroke [15,16,17]. These medications include SGLT2 inhibitors, GLP-1 agonists (particularly liraglutide and semaglutide), and GLP-1 agonists combined with glucose-dependent insulinotropic polypeptide (GIP), such as tirzepatide.

Importantly, these medications show even more significant beneficial effects in diabetic patients, although the mechanism of action is not yet fully understood. They may promote an anti-inflammatory effect. Recent trials and meta-analyses involving either canakinumab or colchicine underline the role of anti-inflammatory agents in preventing cardiovascular events [18,19,20,21]. Therefore, a low dose of colchicine should be considered for patients with chronic coronary syndrome. This represents another innovation compared to previous guidelines.

Lipid-lowering treatments, including statins, ezetimibe, or PCSK-9 inhibitors, are highly recommended based on tolerability and target achievement. For patients who are intolerant to statins, bempedoic acid should be considered [22,23]. Notably, the 2024 guidelines strongly recommend using ACE inhibitors over angiotensin receptor blockers (ARBs) to reduce the progression of atherosclerosis and related events, such as myocardial infarction and stroke, regardless of the left ventricular ejection fraction [24,25,26,27,28,29]. In cases where angina due to non-obstructive coronary artery disease and ischaemia with no obstructive coronary artery disease results from endothelial dysfunction, ACE inhibitors are also recommended due to their anti-apoptotic effects on the endothelium of coronary arteries [26,30]. This marks an important innovation in treatment.

As expected, the guidelines align with previous recommendations regarding antiplatelet and/or antithrombotic therapies for patients with chronic coronary syndrome or those following a myocardial infarction, percutaneous coronary intervention (PCI), or coronary artery bypass grafting (CABG). The duration of dual antiplatelet therapy (DAPT) after PCI depends on the ischaemic/bleeding risk ratio, ranging from 1 to 12 months, with 6 months being the recommended duration. Anticoagulants, including direct oral anticoagulants (DOAC) or vitamin K antagonists, are typically not indicated unless the patient also has atrial fibrillation (AF), in which case, anticoagulation is necessary for stroke prevention. Triple antithrombotic therapy may be considered for AF patients undergoing PCI, but it should not exceed 1 to 4 weeks, followed by DAPT. However, current evidence suggests that double antithrombotic therapy with direct oral anticoagulants and clopidogrel is preferable [30].

The 2024 guidelines propose several new pharmacological options for managing risk factors and preventing adverse events. A critical issue remains regarding adherence to health behaviours and pharmacological treatments. Poor medication adherence is a significant barrier to successful prevention, with studies indicating that 40–60% of patients prescribed cardiovascular medications do not follow their prescribed regimens. The guidelines express deep concern about adherence and recommend various strategies to improve it. These strategies include addressing psychological aspects, assisting in maintaining adherence, collaborating with patients to overcome practical challenges, and utilising fixed-dose combinations.

The guidelines strongly advocate for using mobile health interventions and emerging technologies, such as wearable devices and ingestible sensors monitoring medication absorption. Regardless of technological advances, the fundamental principle remains: successful medication adherence requires minimising the number of pills taken, emphasising single-pill combinations, and fostering understanding, support, and personalised strategies that fit into patients’ lives.

Overall, these guidelines present a clear and comprehensive set of recommendations, particularly in managing risk factors above those presented by the previous GLs [31]. The emphasis on medication adherence reinforces the idea that the most effective treatment regimen is one that patients can consistently follow;

- Antianginal/Anti-Ischemic Pharmacological Therapy

This section is brief, with several aspects deferred to the supplementary data. We were unsurprised, as there is no new information, no evidence that any antianginal medication improves prognosis, and no head-to-head trials comparing the historical first-approved antianginal agents with the most recent ones. Paradoxically, the newer antianginal drugs, which were classified as second- or third-line in the previous guidelines [32], have more contemporary evidence supporting their use than traditional first-line drugs, such as nitrates, beta-blockers (BB), and calcium antagonists [33].

**Figure 2:**
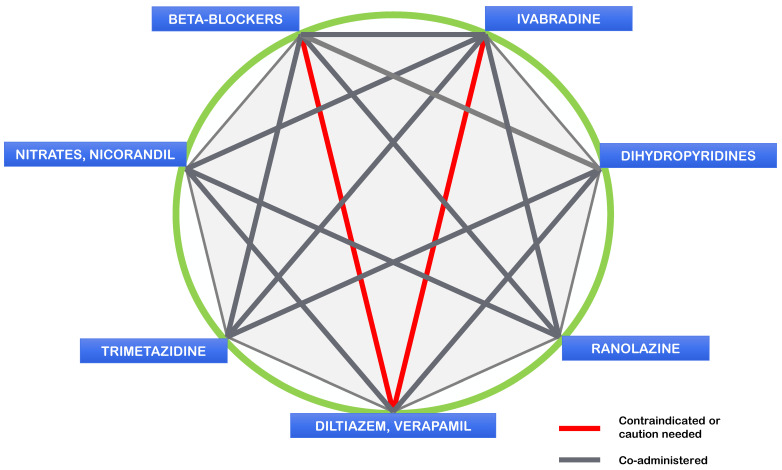
The Diamond Approach: possible combination of classes of antianginal drugs. Adapted from Ferrari et al. [2]. In the heptagon, all the vertices represent one of the available categories of antianginal drugs. Red lines represent the contraindicated associations, while the grey lines represent the possible and useful associations.

A recent systematic meta-analysis covering 50 years of medical treatment for chronic coronary syndromes (CCSs) has shown that early studies were conducted with small sample sizes and used different endpoints than those currently required for registration by European and American agencies, employing outdated analysis methods [33,34,35]. Based on the information available at their registration, nitrates, beta-blockers, and calcium antagonists could not be approved for the treatment of CCSs. The overall evidence indicates that no antianginal drug is superior to another, and equivalence has been established only among three drug classes: beta-blockers, calcium blockers, and ivabradine. This is why, in 2018, the “Diamond Approach to the treatment of angina” was proposed and widely adopted [2]. A revised graphical illustration of the original Diamond Approach is provided below (Figure 2).

**Figure 3:**
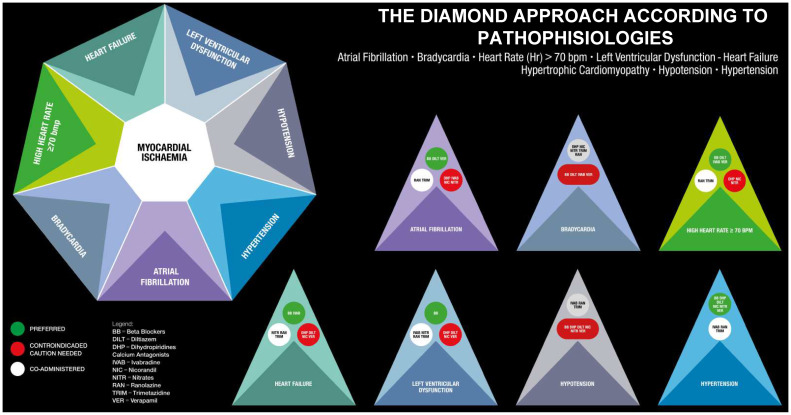
The Diamond Approach according to pathophysiologies. Each situation needs a specific approach without classification of drugs as first and second choices but listing them all at the same level.

**Figure 4:**
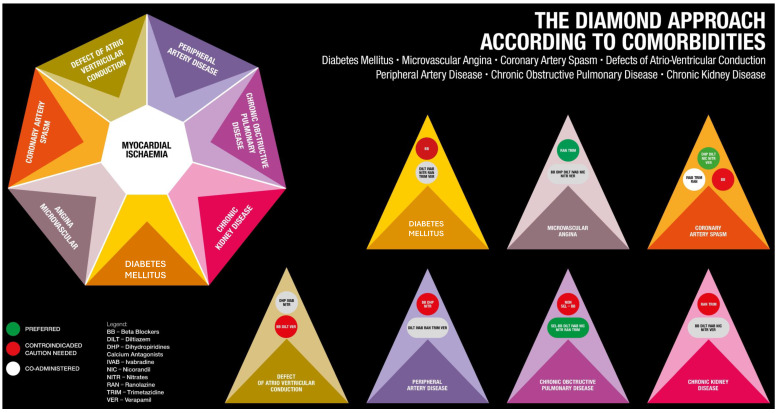
The Diamond Approach according to comorbidities. Each situation needs a specific approach without classification of drugs as the first and second choices but listing them all at the same level.

In alignment with the central theme of the current guidelines, an individualised approach to treating angina is suggested, considering the patients, their comorbidities, and the underlying mechanisms of the disease. All the different possibilities of drug treatment are also summarised in Figure 3 and Figure 4 according to pathophysiologies and comorbidities. It is interesting to note that no antianginal drug has shown any prognostic benefit; conversely, event-reducing drugs have no antianginal effects, either. So, apart from combining a haemodynamic agent and a metabolic agent to begin with, to address the possible multiple pathophysiologies involved in each case and choose agents suitable or contraindicated for special situations, there is very little to choose from between the agents. However, patient preference and availability and cost issues are also to be considered.

These considerations are indeed the basis of the idea of the Diamond Approach and its graphic. Instead of a traditional hierarchical vertical order with first, second, and third choices, all antianginal drugs are graphically represented on the same circular or octagonal line, resembling a diamond. Per the previous guidelines, it seems unscientific to designate older drugs as first-line treatments [25,29]. The diamond scheme allows for the recommendation of the most appropriate agent for each underlying pathophysiology or comorbidity based on the auxiliary properties of each drug class, in addition to their antianginal and anti-ischaemic actions. The diamond graphic also indicates the most helpful and contraindicated combinations [2] (Figure 1).

The 2024 guidelines have made significant progress by graphically adopting the Diamond Approach, which emphasises the treating physician’s responsibility to select the most appropriate medications based on individual patient needs, like the approach in managing arterial hypertension. The diamond scheme is presented clearly and helpfully, offering recommendations for drug selection according to physiological and comorbidity considerations. Notably, this is a departure from previous guidelines, which outlined a fixed, stepwise sequence of drug treatments [32,36].

However, while the graphic representation aligns with the Diamond Approach, the accompanying text and tables present a traditional hierarchical approach. These sections still indicate first-line treatments (short-acting nitrates, beta-blockers, and calcium antagonists) and second-line options (long-acting nitrates, nicorandil, ranolazine, ivabradine, and trimetazidine). This discrepancy suggests that although the update is a step in the right direction, it has not fully embraced the Diamond Approach’s original intent.

Additionally, the evidence level for beta-blockers and calcium antagonists has been downgraded from level A to level B, which is concerning given the lack of supporting evidence for this change. This decision indicates a shift in belief from the previous committee. In scenarios where angina is not adequately controlled, the current guidelines recommend using beta-blockers and calcium antagonists in combination despite clinical trials showing no significant additional benefits from combining several antianginal medications with haemodynamic effects [37,38,39]. These statements and, in particular, the grading in different levels, are clearly in contrast with the “*physiology*” of the authors of the consensus statement of the Diamond Approach (*2*). If there is no evidence that one drug is superior to any other, on which basis should it be offered according to the guidelines without a higher level of evidence compared to the others? This is against the principle that the guidelines should be written and classify drugs according to the evidence.

Some recommendations raise further questions. For instance, ivabradine is indicated as an add-on therapy for patients with left ventricular systolic dysfunction (ejection fraction [EF] > 40%) but is contraindicated for those with EF > 40% without clinical heart failure. This stance contradicts the European Medicines Agency’s guidance [40] and findings from several large trials, such as BEAUTIFUL [41] and its angina subgroup analysis [42], along with ASSOCIATE [43,44]. Furthermore, trimetazidine has been downgraded from Class IIa to IIb with no new evidence. While beta-blockers are recommended as first-line treatment for patients with ANOCA/INOCA to manage microvascular angina and reduced blood flow, this recommendation lacks supportive studies. It raises the question of why alternatives like verapamil or diltiazem, which also reduce oxygen consumption and improve coronary flow, were not considered. Similarly, drugs such as trimetazidine and ranolazine, which act at the cardiac cell level, could be viable options. These recommendations reflect the experts’ opinions rather than being based on solid evidence.

When the GL committee was questioned during the ESC congress in London about the rationale behind these changes, they mentioned aligning with the American guidelines [45]. In the United States, ivabradine is indicated for heart failure, while trimetazidine is not available. However, the ESC guidelines are intended for practising physicians in Europe, and it is hoped that they will uphold their own independent beliefs in the absence of new scientific data. In addition, several recent studies show that recurrent or persistent angina occurs despite invasive and/or medical therapies. The use of two haemodynamic agents is often not enough to reduce angina attacks in symptomatic patients, and the availability of drugs that improve anaerobic metabolism (*like trimetazidine*) might be important.

The section on combination therapy and the associated suggestions in the diamond graphic are promising and forward-thinking. Although specific studies on fixed-dose combinations of antianginal drugs akin to those in hypertension are yet to be conducted, this approach could represent a significant advancement in the future.

In conclusion, adopting the Diamond Approach marks a noteworthy improvement over previous guidelines. While we appreciate this development, it could have been accompanied by a more comprehensive explanation of its underlying principles.

## 5. Myocardial Revascularisation in Patients with CCS

This section thoroughly overviews all existing trials and registries related to CABG (coronary artery bypass grafting) and PCI (percutaneous coronary intervention). As anticipated, there are no significant innovations; however, there is a general trend towards being more cautious with indications for intervention, which are now limited to severe left main and three-vessel disease, particularly when accompanied by left ventricular dysfunction.

Implementing disease-modifying agents and using the most accurate diagnostic procedures for assessing ischaemia, as recommended by the 2024 guidelines, will further help restrain unnecessary revascularisation indications. These decisions should be made collaboratively between patients and healthcare professionals, ideally involving a multidisciplinary heart team that includes anaesthetists and other specialists. Lessons from the ISCHEMIA, REVIVED-BCIS2, and a recent Korean National Insurance registry suggest a less intervention-centric approach to managing CCS or ischaemic cardiomyopathy [9,10,11,12,13,46,47].

The guidelines also suggest incorporating institutional protocols developed by the heart team into standard practice, which could be beneficial from a legal standpoint. Sections discussing virtual percutaneous interventions, which combine results from CCTA (coronary computed tomography angiography) with FFR (fractional flow reserve), LFR (local flow reserve), or QFR (quantitative flow ratio), as well as hybrid revascularisation methods that combine minimally invasive surgery on the LAD (left anterior descending artery) with PCI for other arteries, are noteworthy. However, the frequency of these appealing options needs to be higher, with only a few data points available.

Regarding the complex decision of revascularisation in patients with unprotected left main lesions, the current guidelines reaffirm the recommendations of the previous 2022 joint ESC/EACTS guidelines stating that CABG is preferred over PCI due to its lower risk of spontaneous myocardial infarction (MI) and the lesser need for repeat revascularisation. In low-complex stenosis, defined as a syntax score of ≤22, PCI is favoured over CABG [48,49,50]. In addition, the guidelines take particular care regarding the “*voice of the patients*”, as a diagnosis of CCS impacts their daily and long-term lives, particularly when there is uncertainty about the optimal strategy to follow. Shared decision-making, not only among professionals but also between them and patients, is itself part of the therapy, as patients feeling fully confident and involved is central to their future care.

This section is an excellent and comprehensive continuation of the previous guidelines’ recommendations.

## 6. Treatment of Specific Groups of Patients

The final section of the guidelines analysis provides a detailed examination of the occurrence of chronic coronary syndromes in patients with pre-existing comorbidities, such as heart failure (HF), valvular diseases, atrial fibrillation (AF), rheumatic diseases, and cancer. Assessing CCS in these patients is crucial yet can be challenging, particularly in cases of heart failure where CCS may be underestimated. Functional imaging is preferred for patients with heart failure with preserved ejection fraction (HFpEF), especially when there is a suspicion of ischaemia with no obstructive coronary artery disease. Concurrently, CCTA is recommended for patients suspected of having epicardial stenosis.

In terms of treatment, calcium antagonists, particularly verapamil and diltiazem, should be avoided as they may increase heart failure-related events. Other antianginal medications can be administered, and some, such as beta-blockers and ivabradine, are indicated for treating heart failure with reduced ejection fraction (HFrEF). Similar recommendations apply for HFpEF, where agents like ACE inhibitors, SGLT2 inhibitors, and GLP-1 agonists are already suggested. Revascularisation in patients with severe heart failure presents additional challenges, often requiring left ventricular assistance and mechanical cardiac support. The guidelines were released before the data on obesity with SGLT_2_i and GLP_1_ activators on HF with preserved ejection fraction (*HFpEF*) became available. It follows that these classes of drugs need to be considered for the treatment of the two conditions, CCS and HFpEF, and prevention of the progression of atherosclerosis.

Additionally, the guidelines further explore CCS in patients with non-obstructive coronary arteries, particularly highlighting the significant number of patients (mainly women) suffering from angina and/or ischaemia with no obstructive coronary artery disease. Ischaemia in these patients may stem from excessive vasoconstriction (spasm) or microvascular dysfunction. In cases of epicardial spasm, the administration of acetylcholine (ACH) at various dosages is recommended for diagnostic purposes, following six identifiable endotypes: 1. endothelial dysfunction (a specific test); 2. altered vasodilation (a low coronary flow reserve [CFR] and a high index of microvascular resistance [iMR]); 3. epicardial spasm; 4. microvascular vasospastic angina (a low dose of ACH); 5. a combination of all endotypes; and 6. an equivocal response. This comprehensive analysis aims to enhance the understanding and management of these complex cardiovascular conditions.

However, this approach is challenging because it is time-consuming for busy catheterisation laboratories, requires significant experience, and cannot be implemented everywhere. While it is appealing, it is impractical except in dedicated research centres. In line with the underlying theme of the guidelines and the original Diamond Approach, recognising the endotypes is essential for treatment (Figure 3 and Figure 4). For instance, in cases of epicardial or microvascular spasms, beta-blockers are contraindicated. This contrasts with the general recommendation of using beta-blockers as the first-line treatment for angina with no obstructive coronary arteries, as indicated in the table but not in the figure.

Calcium antagonists and nitrates, on the other hand, are highly recommended. At the same time, drugs aimed at improving cardiac metabolism (trimetazidine) or inhibiting the late sodium current in cardiac cells (ranolazine), can be considered even though there is no definitive evidence supporting their use. Microvascular dysfunction is another cause of ANOCA/INOCA. In these patients, particularly when persistently symptomatic and with poor quality of life, invasive coronary functional testing is recommended to identify the different possible endotypes. A transthoracic doppler of the LAD stress echocardiography, CMR, and PET are alternative, non-invasive options. Ischaemia may result from structural or functional alterations of the microcirculation, which lead to impaired coronary flow reserve and reduced vasodilatory capacity, whether endothelial-dependent or independent.

A complex and invasive diagnostic algorithm is summarised in a figure, along with specific treatment recommendations; however, these recommendations are not evidence-based as studies do not support them. The treatment of microvascular angina caused by abnormal vasodilation includes all antianginal drugs, not just beta-blockers, with a new recommendation for ACEi due to their beneficial effects on the endothelium. ACEi and statins are also indicated in the presence of coexisting atherosclerosis. It is important to note that the suggested therapeutic interventions have never been tested in specific endotypes, apart from using calcium antagonists and nitrates in epicardial vasospastic angina. Thus, the recommendations largely reflect the beliefs of the proponents.

Current studies testing specific therapies for INOCA/ANOCA are duly reported, including the WARRIOR trial [51], which examines the combination of intensive statin/ACEi/angiotensin receptor blocker (ARB) treatment in women diagnosed with ANOCA, and the PRIZE trial [52], which involves zibotentan, an endothelin receptor antagonist aimed at counteracting endothelial-mediated vasoconstriction.

Adding a section on INOCA/ANOCA is justified, as angina without obstructive coronary arteries is associated with the same, if not higher, mortality rates, increased hospitalisations, poorer quality of life, and more significant physical and mental stress compared to epicardial angina [53,54,55,56].

In summary, a focused section on specific comorbidities is helpful. The careful attention given to ANOCA/INOCA, which presents significant challenges in diagnosis and treatment, represents a valuable innovation.

## 7. Conclusions

One hundred and twenty-three pages and one thousand two hundred references are a significant amount of information to read and remember. Nevertheless, it is the duty of the guidelines to be comprehensive and report all available data. The guidelines reflect the complexity and ongoing evolution of understanding, diagnosing, and treating diseases. The 2024 Guidelines on CCS present this information clearly, which, wherever possible, is innovatively aided by engaging illustrations. As mentioned earlier, this represents a notable improvement over the previous version, which had numerous missteps in its recommendations for medical treatment.
